# Imatinib-Induced Changes in Anxiety-Like Behaviors are Associated With Prefrontal Cortical Metabolic Remodeling in Mice

**DOI:** 10.31083/AP51694

**Published:** 2026-08-17

**Authors:** Jieyu Ji, You Wang, Shanglan Qu, Xin Peng, Ruixue Ma, Hanwei Li, Jinlong Chang, Lijuan Li, Yanjiao Li, Zhiting Gong

**Affiliations:** ^1^School of Medicine, Dali University, 671003 Dali, Yunnan, China; ^2^School of Pharmacy, Faculty of Health and Medical Sciences, Taylor’s University, 47500 Subang Jaya, Selangor, Malaysia

**Keywords:** tyrosine kinase inhibitors, anxiety, imatinib, prefrontal cortex, metabolomics, sphingolipids

## Abstract

**Background:**

Anxiety-related symptoms are frequently reported in patients with chronic myeloid leukemia (CML) receiving tyrosine kinase inhibitor (TKI) therapy. However, the role of TKIs, including imatinib, in development of anxiety remains unclear. The prefrontal cortex (PFC) is closely associated with anxiety-like behaviors, and its metabolic components, particularly lipid- and myelin-related metabolites, are involved in their regulation. Whether imatinib affects brain metabolism remains unknown. This study aimed to evaluate the effects of imatinib on anxiety-like behaviors and to investigate associated metabolic alterations in the PFC.

**Methods:**

Anxiety-like behaviors were assessed using a battery of behavioral assays in imatinib-treated mice. PFC tissues from imatinib-treated and control mice were subjected to untargeted metabolomic analysis.

**Results:**

Imatinib-treated mice exhibited selective alterations in anxiety-like behaviors. Metabolomic analysis revealed distinct metabolic profiles between the two groups, with significant changes in lipid-related pathways, particularly sphingolipid metabolism. These findings extend the current understanding of the metabolic basis of anxiety-like behaviors and suggest that TKI treatment may influence neuropsychiatric processes through metabolic remodeling of the PFC.

**Conclusions:**

This study highlights a potential link between TKI therapy and brain metabolism and provides a framework for understanding the neurobiological basis of anxiety-related effects. These findings may inform future research on the neuropsychiatric consequences of targeted cancer therapies.

## Main Points

1. Imatinib treatment in mice demonstrates anxiolytic-like behavioral effects.

2. Imatinib causes significant alterations in the metabolic profile of the prefrontal cortex.

3. The proposed mechanism underlying the potential link between metabolic remodeling and behavioral changes.

## 1. Introduction

Anxiety is one of the most frequently reported psychological symptoms in patients with chronic myeloid leukemia (CML). Previous clinical evidence has shown that mental health disturbances, particularly anxiety, are highly prevalent among CML patients [1]. Assessments using standardized instruments, including the Patient Health Questionnaire-9 (PHQ-9), the Generalized Anxiety Disorder-7 (GAD-7), and the Impact of Event Scale-Revised (IES-R), have demonstrated that CML is significantly associated with elevated risks of anxiety, depression, psychological distress, and hyperarousal, indicating a substantial psychological burden in this patient population [2].

Anxiety is also commonly observed among CML patients receiving tyrosine kinase inhibitor (TKI) therapy [3,4]. Epidemiological studies have reported that approximately one-fifth to one-quarter of TKI-treated CML patients experience anxiety symptoms, ranging from mild to moderate or severe levels [4]. Furthermore, anxiety and depressive symptoms have been shown to be more prevalent in specific subgroups of patients, including females, individuals with lower educational attainment, patients with multiple comorbidities, those receiving second- or third-line TKI therapy, and patients with higher treatment-related financial burden [5]. These findings collectively highlight the high prevalence of anxiety in CML patients undergoing TKI treatment and suggest that both clinical and socioeconomic factors may contribute to its development.

In addition, psychological interventions, including mindfulness-based approaches, have been reported to improve quality of life and alleviate anxiety, depression, fatigue, pain, and other treatment-related symptoms in TKI-treated CML patients [6]. Moreover, accumulating evidence suggests that common adverse effects of TKI therapy—such as fatigue, gastrointestinal discomfort, recurrent infections, musculoskeletal pain, abdominal pain, and appetite loss—are closely associated with increased anxiety symptoms [7,8]. Together, these observations support a close relationship between anxiety and TKI-related treatment. Importantly, there is emerging evidence indicating that TKI therapy itself may contribute to the onset or exacerbation of anxiety symptoms. Case reports and clinical observations have described worsening anxiety and sleep disturbances following initiation of specific TKIs, raising concerns regarding the potential neuropsychiatric effects of these agents [9]. Taken together, anxiety in CML patients appears to be closely linked to TKI treatment and its associated adverse effects, underscoring the importance of addressing mental health as part of comprehensive CML management.

Currently, several TKIs are approved for the treatment of CML, including imatinib, dasatinib, nilotinib, and bosutinib [10,11]. Among these agents, imatinib is the first-generation and most extensively used TKI, specifically targeting the breakpoint cluster region–Abelson proto-oncogene 1 (BCR-ABL1) fusion tyrosine kinase that drives CML pathogenesis [12,13]. Since its introduction into clinical practice in 2001, imatinib has dramatically improved survival outcomes and long-term prognosis in patients with Philadelphia chromosome-positive CML [14,15]. Despite the availability of second-generation TKIs, imatinib remains the standard first-line therapy for many adult CML patients due to its well-established efficacy, safety profile, and widespread clinical use [16,17]. Therefore, focusing on imatinib in the present study reflects real-world treatment practice and has substantial clinical relevance.

Anxiety-like behaviors have been closely linked to metabolic alterations in the cerebral cortex, particularly the prefrontal cortex, which plays a central role in emotional regulation [18,19,20]. Multiple neurotransmitter systems and metabolic pathways are involved in the modulation of anxiety-related behaviors [21]. The γ-aminobutyric acid (GABA) ergic system, the principal inhibitory neurotransmitter system in the brain, has been extensively implicated in anxiety regulation, with disruptions in GABA synthesis or receptor function leading to increased anxiety-like phenotypes in animal models [22,23]. Serotonergic signaling, particularly involving the 5-hydroxytryptamine 1A (5-HT1A) receptor, has also been shown to interact with cortical excitatory–inhibitory balance and influence anxiety-related behaviors [24,25]. In addition to classical neurotransmitter systems, increasing evidence suggests that broader metabolic processes, particularly lipid metabolism and myelin-related pathways, play critical roles in maintaining neuronal function and emotional regulation [26,27,28]. Alterations in lipid composition can affect membrane fluidity, receptor function, and synaptic transmission, while myelin integrity is essential for efficient neural signaling within the prefrontal circuits [29]. Disruption of these processes has been associated with emotional dysregulation and anxiety-related phenotypes in both clinical and preclinical studies.

In addition, systemic metabolic and immune-related alterations have been reported to affect brain monoamine levels and emotional behaviors, highlighting the interplay between peripheral metabolism and central nervous system function [30]. This peripheral–central interaction suggests that systemic metabolic perturbations may influence brain function through multiple pathways, including blood–brain barrier transport, neuroinflammation, and metabolic signaling, thereby contributing to behavioral alterations [31,32]. Changes in lipid and sphingolipid metabolism have also been associated with anxiety- and depression-like behaviors in rodent models [33]. Notably, these metabolic alterations may also intersect with myelin-related processes, further linking metabolic remodeling to functional changes in neural circuits involved in anxiety regulation [34,35,36]. Collectively, these findings indicate that anxiety-related behaviors are accompanied by broad metabolic alterations in the brain, involving multiple neurotransmitter systems and metabolic pathways.

Taken together, these clinical and experimental observations highlight a potential link between TKI exposure, metabolic alterations, and anxiety-related behaviors, underscoring the need for a system-level approach. Metabolomics provides a powerful and unbiased platform to capture such global changes and to identify candidate pathways for further mechanistic investigation. In the present study, we employed behavioral assays combined with untargeted metabolomic analysis of the prefrontal cortex to systematically characterize the relationship between imatinib exposure, anxiety-like behaviors, and prefrontal cortical metabolic remodeling, and to identify candidate pathways for future mechanistic investigation.

## 2. Materials and Methods

In this study, we employed a combination of behavioral assays and untargeted metabolomic analysis to investigate the relationship between imatinib treatment, anxiety-like behaviors, and metabolic alterations in mice. Behavioral tests, including the open field test, light–dark box test, and elevated plus maze, were used to assess anxiety-like phenotypes. Untargeted LC–MS-based metabolomics was performed to capture global metabolic changes in the prefrontal cortex. This integrated approach enables a system-level characterization of behavioral and metabolic alterations.

### 2.1 Animals

C57BL/6J mice were purchased from Speyford (Beijing) Biotechnology Co., Ltd. All animals used in this study were 8–10 weeks of age. Mice were housed at the Experimental Animal Research Center of Dali University under standard laboratory conditions, with a 12-h light/12-h dark cycle and an ambient temperature maintained at 18–22 °C. Food and water were provided ad libitum throughout the study.

All experimental procedures were designed to minimize animal suffering. For each experiment, mice were selected from the same cohort of age-matched male animals. At the experimental endpoint, mice were deeply anesthetized with 1% sodium pentobarbital (50 mg/kg, intraperitoneally). Adequate depth of anesthesia was confirmed by the absence of pedal withdrawal and corneal reflexes. Mice were then euthanized by decapitation, and brain tissues were rapidly collected. The prefrontal cortex was dissected, snap-frozen in liquid nitrogen, and stored at −80 °C until metabolomic analysis. 

### 2.2 Drug Administration and Model Establishment

C57BL/6J mice were randomly assigned to either a control group or an imatinib-treated group. Imatinib (HY-15463, MedChemExpress LLC, Monmouth Junction, NJ, USA) was incorporated into the standard chow at a concentration calculated to achieve an estimated daily intake of 200 mg/kg/day, based on average body weight and food consumption. Control mice received standard chow without drug supplementation. The treatment period lasted for 14 consecutive days.

### 2.3 Behavioral Tests

A series of behavioral assays, including the open field test and the light–dark box test, were performed to assess whether imatinib treatment induced anxiety-like behaviors in mice.

#### 2.3.1 Open Field Test (OFT)

The open field apparatus [37] consisted of a square arena measuring 45 × 45 × 30 cm, with an illumination level of approximately 150 lux. Mice were transferred from the housing room to the testing room and allowed to habituate for at least 1 h prior to testing. For each trial, mice were individually placed into the center of the open field arena. Locomotor activity was recorded for 15 min. Behavioral parameters, including total distance traveled and time spent in the center zones, were analyzed using VisuTrack software (Version 3.0, Shanghai XinRuan Information Technology Co., Ltd., Shanghai, China). The open field was cleaned with 75% ethanol between animals to eliminate olfactory cues.

#### 2.3.2 Light–Dark Box Test (LDBT)

Anxiety-like behavior was assessed using the light–dark box test [38]. The apparatus consisted of two equal-sized compartments connected by a small opening. The light compartment (30 × 30 × 30 cm for mice) was white and brightly illuminated (150 lux), whereas the dark compartment was black and enclosed with an opaque cover.

Mice were transferred from the housing room to the testing room and allowed to habituate for at least 1 h prior to testing. The mouse was placed in the white area, and then the connecting door was opened, allowing free exploration between the light and dark compartments for 10 min. Behavioral parameters, including the number of transitions between compartments and the time spent in each compartment, were recorded and analyzed using VisuTrack behavioral analysis software (Version 3.0, Xinruan, Shanghai, China).

#### 2.3.3 Elevated Plus Maze (EPM)

The elevated plus maze (EPM) [39] consisted of two opposing open arms (36 × 6 cm) and two opposing closed arms (36 × 6 × 15 cm), connected by a central platform (6 × 6 cm). The maze was elevated 50–60 cm above the floor, and the illumination level was maintained at approximately 150 lux. Prior to testing, mice were transferred from the animal housing room to the testing room and allowed to habituate for at least 1 h. Each mouse was placed individually in the center of the maze, facing an open arm, and allowed to freely explore the apparatus. After each trial, the maze was cleaned with 75% ethanol to eliminate olfactory cues.

### 2.4 Metabolomics Detection

#### 2.4.1 Sample Collection

Metabolites are prone to oxidation and degradation; therefore, all experimental procedures must be performed on ice or at low temperatures.

#### 2.4.2 Sample Collection and Storage

Based on the research background and experimental design, PFC brain tissue samples were collected, washed with phosphate-buffered saline (PBS), and centrifuged at 4 °C to obtain cell pellets. The pellets were then transferred to microcentrifuge tubes and stored at −80 °C until further analysis.

#### 2.4.3 Metabolite Extraction

Approximately 50 mg of solid tissue sample was placed in a 2 mL microcentrifuge tube containing a 6 mm diameter grinding bead. A total of 400 µL of extraction solution was added to facilitate metabolite extraction. The sample suspension was homogenized in a cryogenic tissue homogenizer for 6 minutes, followed by low-temperature sonication for 30 minutes. Subsequently, the sample was incubated at −20 °C for 30 minutes, centrifuged for 15 minutes at 4 °C, and the supernatant was transferred to an autosampler vial with an internal insert for liquid chromatography–mass spectrometry (LC–MS) analysis.

#### 2.4.4 Quality Control (QC) Sample Preparation

Quality control samples were prepared by pooling 20 µL aliquots from each individual sample. LC–MS analysis was performed using a Thermo Fisher UHPLC–Q Exactive HF–X system (Thermo Fisher Scientific, Waltham, MA, USA). After separation on an ACQUITY UPLC HSS T3 column (100 mm × 2.1 mm i.d., 1.8 µm; Waters, Milford, MA, USA), the eluate was subjected to mass spectrometric detection. Mobile phase A was water/acetonitrile (95/5, v/v) with 0.1% formic acid, and mobile phase B was acetonitrile/isopropanol/water (47.5/47.5/5, v/v/v) with 0.1% formic acid. The flow rate was 0.40 mL/min, the injection volume was 3 μL, and the column temperature was 40 °C. Mass spectrometry signals were collected in both positive and negative ion scan modes, with a mass scanning range of m/z 70-1050. Ion spray voltage: +3500 V for positive ions and -3500 V for negative ions, the sheath gas was 50 arb, auxiliary heating gas was 13 arb, the ion source heater temperature was 425 °C, and the collision energy was cycled through 20-40-60 V.

### 2.5 Metabolomics Data Processing

#### 2.5.1 Raw Data Processing

Following LC–MS analysis, the raw data were imported into Progenesis QI (Waters Corporation, Milford, MA, USA) for data processing, including baseline correction, peak identification, peak integration, retention time alignment, and peak alignment [40]. This processing generated a data matrix containing retention time, mass-to-charge ratio (*m/z*), and peak intensity values. Metabolite annotation was performed by matching the LC–MS/MS data against multiple databases, including the Human Metabolome Database (HMDB; The Metabolomics Innovation Centre, University of Alberta, Edmonton, AB, Canada; http://www.hmdb.ca/) [41], METLIN (The Scripps Research Institute, La Jolla, CA, USA; https://metlin.scripps.edu/) [42], and an in-house metabolite database.

#### 2.5.2 Statistical Analysis

The resulting data matrix was uploaded to the Majorbio Cloud Platform (Majorbio Bio-pharm Technology Co., Ltd., Shanghai, China; https://cloud.majorbio.com) for subsequent statistical analysis. Data preprocessing was performed as follows: missing values were handled using the 80% rule, followed by missing value imputation. To minimize systematic bias introduced by sample preparation and instrumental variation, peak intensity values were normalized using the total sum normalization method, resulting in a normalized data matrix. Variables exhibiting a relative standard deviation (RSD) >30% in QC samples were excluded, and the resulting data matrix was log_10_-transformed for downstream analysis.

Principal component analysis (PCA) and partial least squares discriminant analysis (PLS-DA) were performed on the preprocessed data matrix using the R package ropls (version 1.6.2; Bioconductor Project, https://bioconductor.org/packages/ropls/). Model stability was assessed using 7-fold cross-validation and permutation testing. Significant differential metabolites were identified based on variable importance in projection (VIP) scores and univariate Student's *t*-test *p*-values obtained from the PLS-DA model.

For differential analysis, log_2_ fold change (log_2_FC) between the ima and control groups was calculated. Nominal *p*-values were obtained using two-tailed unpaired *t*-tests and were further adjusted for multiple testing using the Benjamini–Hochberg procedure. As no metabolites met the significance threshold after false discovery rate (FDR) correction (FDR <0.10), metabolites with |log_2_FC| >0.3 and nominal *p* < 0.05 were considered exploratory candidates.

Pathway Enrichment Analysis: Pathway-level changes were evaluated using Quantitative Enrichment Analysis (QEA) in MetaboAnalyst (version 5.0; Xia Lab, McGill University, Montreal, QC, Canada; https://www.metaboanalyst.ca), which leverages quantitative shifts across the complete metabolite abundance matrix rather than relying on predefined feature-level cutoffs. KEGG (KEGG; Kyoto University Bioinformatics Center, Kyoto, Japan; https://www.genome.jp/kegg/) and HMDB pathway librarieswere used for pathway annotation.

### 2.6 Statistical Analysis

Behavioral test data statistical analyses were conducted using GraphPad Prism 10.0 (GraphPad Software, San Diego, CA, USA), and figures were generated using the same software. Data are expressed as mean ± SEM. The normality of data distribution was assessed using the Shapiro–Wilk test. For comparisons between two groups, unpaired Student’s *t*-tests were applied to normally distributed data, while Mann–Whitney U tests were used for data that did not meet the assumption of normality.

## 3. Results

### 3.1 Imatinib Treatment Reduces Anxiety-Like Behavior in Mice

**Fig. 1.**
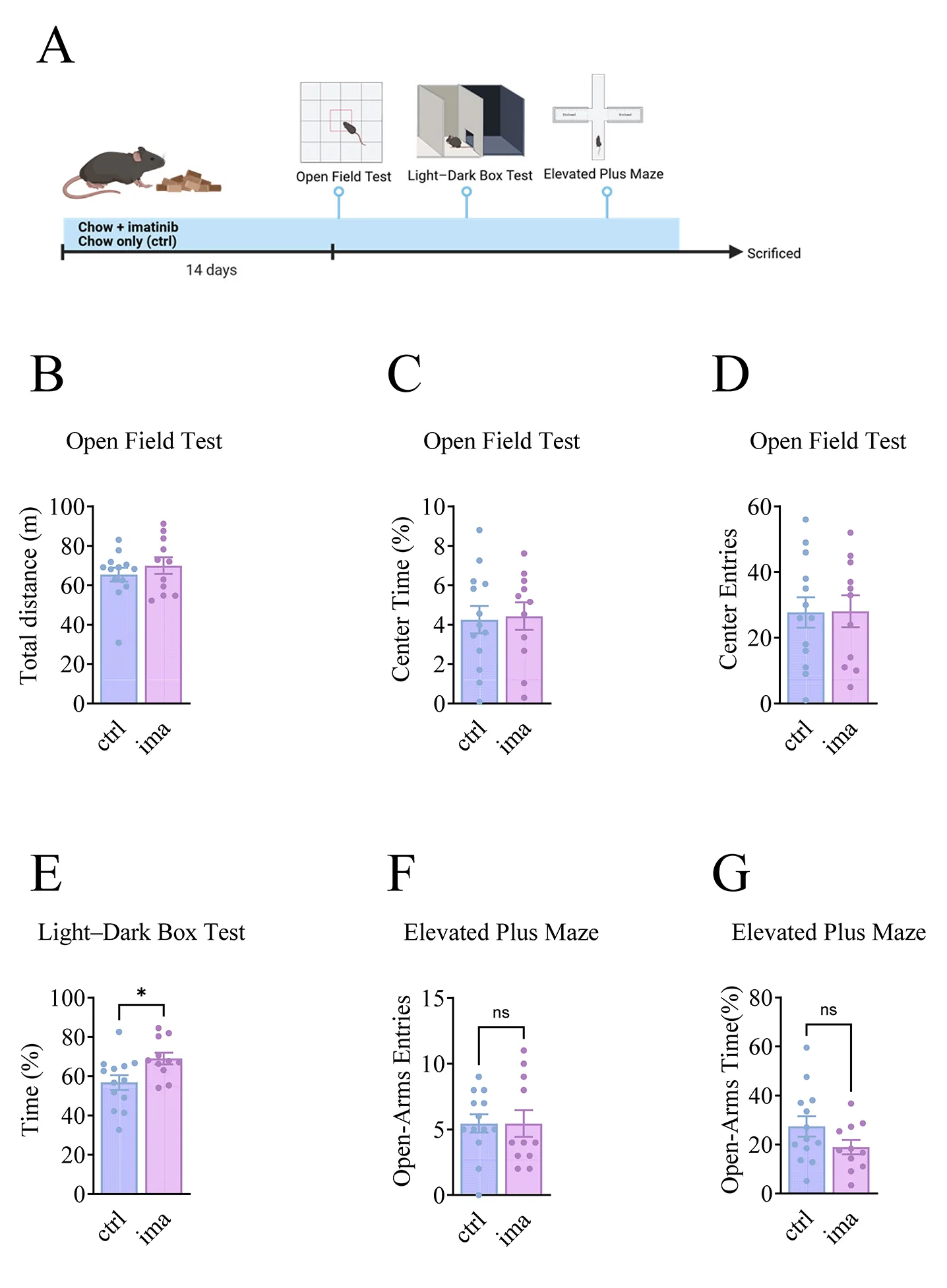
**Behavioral effects of imatinib in anxiety-related tests**. (A) Experimental design. (ctrl, n = 13; ima, n = 11). (B) Total distance traveled in the open field test showed no significant difference between the ima and ctrl groups (Mann–Whitney U test, *p* > 0.05). (C) The percentage of time spent in the center area in the open field test was not significantly different between groups (unpaired *t* test, *p* > 0.05). (D) The number of entries into the center area in the open field test also showed no significant difference between groups (unpaired *t* test, *p* > 0.05). (E) In the light–dark box test, mice in the ima group spent a significantly higher percentage of time in the light compartment compared with controls (unpaired *t* test, *p* < 0.05). (F) The number of open-arm entries did not differ significantly between the imatinib-treated and ctrl groups in the elevated plus-maze test (unpaired *t*-test, *p* > 0.05). (G) The time spent in the open arms did not differ significantly between the imatinib-treated and ctrl groups in the elevated plus-maze test (unpaired *t*-test, *p* > 0.05). ns, not significant; *, *p* < 0.05.

To evaluate the effects of imatinib on anxiety-like behavior, mice were randomly assigned to either a control group (chow only) or an imatinib-treated group (imatinib-containing chow). Behavioral tests were conducted after 14 days of treatment (Fig. 1A). In the open field test, imatinib-treated mice did not show significant differences in total locomotor activity or center exploration compared with control mice (Fig. 1B–D; Tables 1,2), indicating that imatinib treatment did not affect general motor function. The elevated plus-maze did not reveal significant behavioral changes following imatinib administration. Neither the number of open-arm entries nor the time spent in the open arms differed significantly between imatinib-treated and control mice (Fig. 1F–G; Table 3). In contrast, the light–dark box test revealed a significant behavioral change following imatinib administration. Imatinib-treated mice spent a higher proportion of time in the light compartment compared with control mice (Fig. 1E; Table 4), suggesting reduced anxiety-like behavior. Given that time spent in the light compartment is inversely correlated with anxiety levels, these results indicate that imatinib treatment may exert an anxiolytic effect without altering baseline locomotor activity, although this effect appears to be paradigm-dependent.

**Table 1.** **Statistical details of Fig. 1B**.

Comparison of ctrl and ima between groups [mean(SEM)] Mann–Whitney U test				
Groups	ctrl (n = 13)	ima (n = 11)		*p*
Total distance (m)	65.44 (3.497)	70.03 (4.233)		0.5309

**Table 2.** **Statistical details of Fig. 1C,D**.

Comparison of ctrl and ima between groups [mean(SEM)] unpaired *t* test				
Groups	ctrl (n = 13)	ima (n = 11)	*t*	*p*
Center Time (%)	4.256 (0.7027)	4.432 (0.7036)	0.1763	0.8617
Center Entries	27.77 (4.622)	28.09 (4.861)	0.04782	0.9623

**Table 3.** **Statistical details of Fig. 1F,G**.

Comparison of ctrl and ima between groups [mean(SEM)] unpaired *t* test				
Groups	ctrl (n = 13)	ima (n = 11)	*t*	*p*
Open-Arms Entries	5.462 (0.6944)	5.455 (1.012)	0.005840	0.9954
Open-Arms Time (%)	27.37 (4.232)	18.97 (2.949)	1.570	0.1306

**Table 4.** **Statistical details of Fig. 1E**.

Comparison of ctrl and ima between groups [mean(SEM)] unpaired *t* test				
Groups	control (n = 13)	ima (n = 11)	*t*	*p*
Time (%)	56.86 (3.684)	69.02 (3.013)	2.496	0.0205

Taking together, these findings indicate that imatinib produces a context-dependent anxiolytic-like effect, which is preferentially captured by the light–dark box paradigm but not by the open field or elevated plus-maze tests.

### 3.2 Metabolomics Dataset Quality Assessment and Treatment-Associated Metabolic Alterations Following Imatinib Treatment

**Fig. 2.**
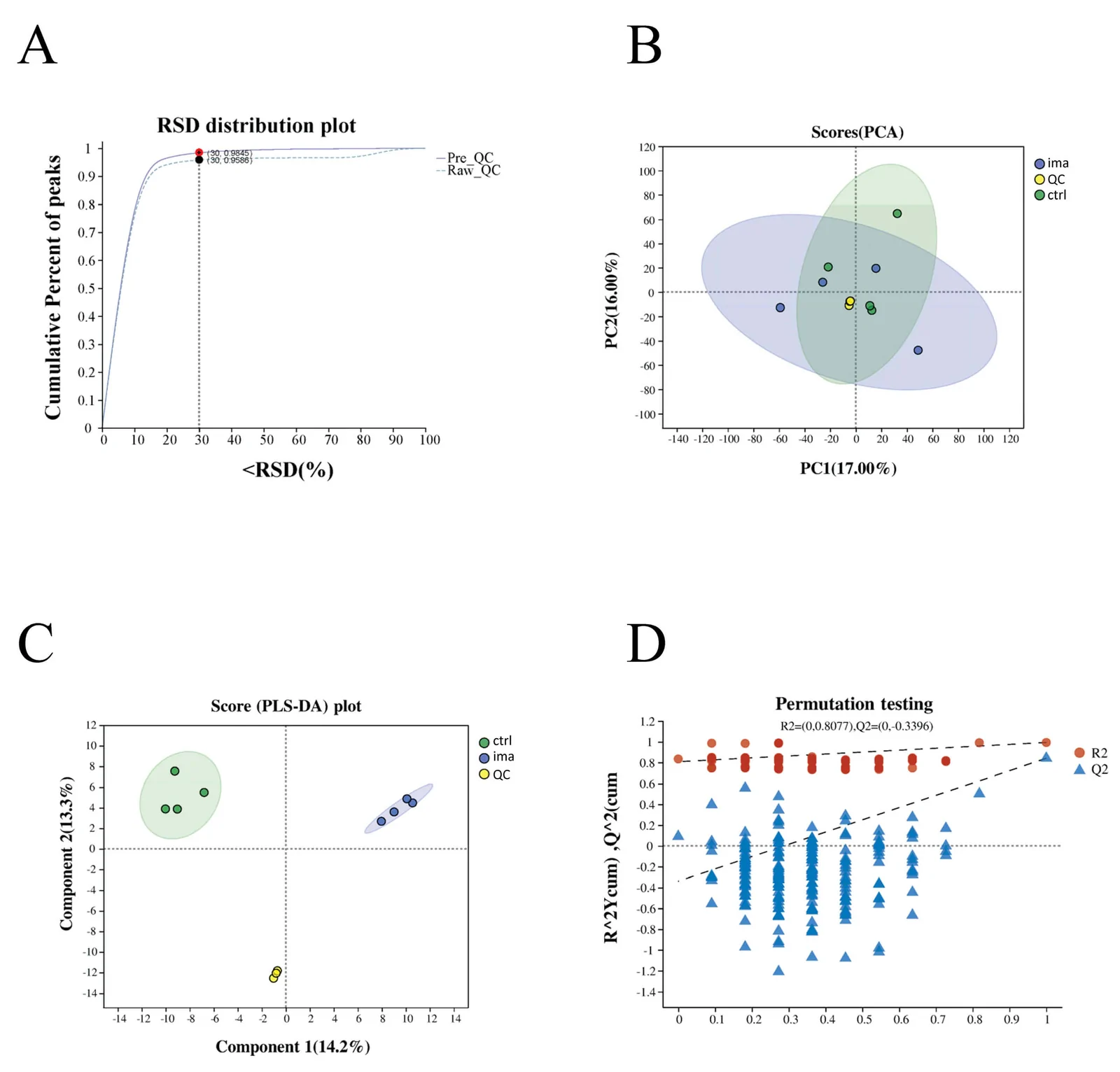
**Quality control and multivariate analysis of the metabolomics dataset**. (A) RSD distribution plot showing the cumulative percentage of detected peaks in QC samples before and after preprocessing. (B) PCA score plot of control (ctrl), imatinib-treated (ima), and QC samples. (C) PLS-DA score plot showing separation between the control and imatinib groups. (D) Permutation test is used to evaluate the reliability of the PLS-DA model. QC, quality control; RSD, relative standard deviation; PCA, principal component analysis; PLS-DA, partial least squares-discriminant analysis.

To investigate whether imatinib treatment alters brain metabolism, untargeted metabolomic profiling was performed on the prefrontal cortex (PFC) of imatinib-treated and control mice. Quality control (QC) assessment demonstrated good analytical stability and reproducibility. The RSD distribution plot showed that the majority of detected metabolites exhibited RSD below 30% in QC samples, indicating acceptable technical variability after data preprocessing (Fig. 2A).

Principal component analysis (PCA) revealed a partial separation between the ima and ctrl groups, while QC samples clustered tightly, further confirming the robustness and reliability of the metabolomics data (Fig. 2B). The first two principal components explained 17.0% and 16.0% of the total variance, respectively.

To further evaluate group discrimination, partial least squares-discriminant analysis (PLS-DA) was performed. Clear separation between the ima and ctrl groups was observed in the score plot (Fig. 2C), suggesting distinct metabolic profiles between groups. Permutation testing indicated that the model was not overfitted, as evidenced by the intercept values (R^2^ = 0.8077, Q^2^ = –0.3396), supporting the reliability of the PLS-DA model (Fig. 2D).

Collectively, these findings indicate that the metabolomics dataset is technically reliable and reflects treatment-associated metabolic alterations, thereby supporting further pathway-based investigation.

### 3.3 Imatinib Treatment Alters the Metabolic Profile of the PFC

**Fig. 3.**
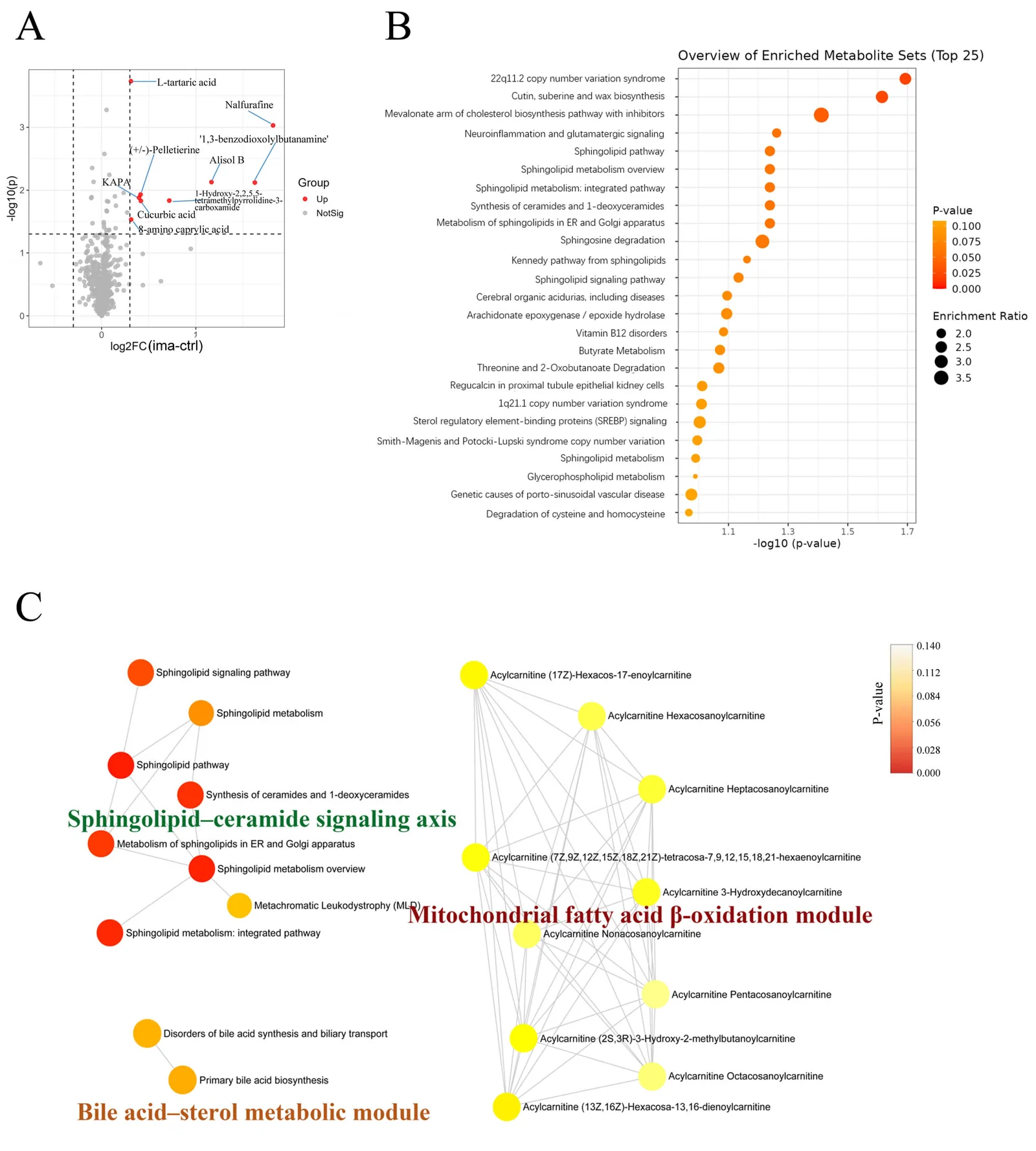
**Differential metabolite analysis and pathway enrichment**. (A) Volcano plot showing differential metabolites between the control and ima groups. Each point represents a metabolite. The x-axis indicates log_2_ fold change (ima vs ctrl), and the y-axis shows −log_10_(*p*-value). Differential metabolites were defined based on |log_2_FC| >0.3 and nominal *p* < 0.05. (B) Pathway enrichment analysis of the differential metabolites. Enriched pathways are ranked based on −log_10_(*p*-value). Dot size represents the enrichment ratio, and color indicates statistical significance. (C) Clustering of enriched metabolic pathways. Network analysis highlights major metabolic modules, including the sphingolipid–ceramide signaling axis, mitochondrial fatty acid β-oxidation module, and bile acid–sterol metabolic module. Nodes represent pathways or metabolites, and edges indicate their associations.

A total of 672 metabolites were annotated across both ionization modes, including 446 detected in positive mode and 226 in negative mode. Differential analysis was performed on all annotated metabolites. No features met the predefined significance threshold of |log_2_FC| >0.3 with FDR <0.10. Given the exploratory nature of this metabolomic investigation, a relaxed criterion (|log_2_FC| >0.3 and nominal *p* < 0.05) was applied to identify suggestive candidates (Fig. 3A). Using this threshold, nine upregulated metabolites were identified, including L-tartaric acid, Nalfurafine, '1,3-benzodioxolylbutanamine', (+/–)-Pelletierine, Alisol B, 8-Amino-7-oxononanoic acid (KAPA), 1-Hydroxy-2,2,5,5-tetramethylpyrrolidine-3-carboxamide, Cucurbic acid, and 8-amino caprylic acid (Fig. 3A). These metabolites as potential candidates associated with treatment-related metabolic alterations and possible modulation of anxiety-like behavior.

Several altered metabolites appeared to be related to lipid-associated metabolic processes. As lipid metabolism plays an important role in neuronal function and inflammatory signaling in the central nervous system [43,44,45], we therefore further examined lipid-related metabolic pathways to better understand the potential metabolic mechanisms underlying the effects of imatinib. Pathway-level enrichment analysis using the Lipidomics library revealed coordinated alterations primarily involving sphingolipid-related pathways, including sphingolipid metabolism and sphingolipid signaling. Additional enrichment was observed in sterol regulatory processes, suggesting remodeling of membrane lipid composition [46]. Notably, pathways linked to neuroinflammation and glutamatergic signaling were also enriched, indicating that lipid metabolic shifts may intersect with neural signaling and inflammatory regulation (Fig. 3B).

Network-based pathway clustering of the metabolomics dataset revealed three major metabolic modules associated with imatinib treatment (Fig. 3C). The first cluster was enriched for pathways involved in sphingolipid metabolism and ceramide synthesis, including sphingolipid signaling, ceramide biosynthesis, and sphingolipid metabolism in the endoplasmic reticulum (ER) and Golgi apparatus, collectively forming a sphingolipid–ceramide signaling axis. A second prominent cluster consisted predominantly of long-chain acylcarnitines, including hexacosanoylcarnitine, heptacosanoylcarnitine, and other very-long-chain acylcarnitine species, which are functionally linked to mitochondrial fatty acid β-oxidation. The tight connectivity among these metabolites suggests coordinated regulation of mitochondrial lipid utilization and energy metabolism. The third cluster was enriched for pathways related to bile acid and sterol metabolism, including primary bile acid biosynthesis and disorders of bile acid synthesis and biliary transport, forming a bile acid–sterol metabolic module.

Together, these results indicate that imatinib treatment is associated with coordinated remodeling of sphingolipid signaling, mitochondrial fatty acid oxidation, and bile acid metabolism, suggesting a metabolic network linking lipid signaling and mitochondrial energy metabolism.

## 4. Discussion

Tyrosine kinase inhibitors (TKIs), including imatinib, have been widely used in the treatment of chronic myeloid leukemia, and increasing evidence suggests that these agents may exert neuropsychiatric effects beyond their anticancer activity. In the present study, we investigated the behavioral and metabolic effects of imatinib treatment in mice. Behavioral analyses demonstrated that imatinib administration produced a selective anxiolytic-like phenotype without affecting general locomotor activity. Specifically, no significant differences were observed between the ima and control groups in total distance traveled, center time, or center entries in the open field test, indicating that imatinib did not alter basal locomotor activity or exploratory behavior. In contrast, mice treated with imatinib spent significantly more time in the light compartment in the light–dark box test, suggesting a reduction in anxiety-like behavior.

The discrepancy among these behavioral paradigms likely reflects differences in the specific anxiety domains measured by each assay. The open field test primarily evaluates locomotor activity and exploratory behavior, whereas the light–dark box paradigm is particularly sensitive to innate aversion to brightly illuminated environments [38]. Therefore, the increased time spent in the light compartment suggests that imatinib may preferentially modulate neural processes involved in innate threat processing rather than broadly affecting anxiety or locomotor activity [47]. These results are generally consistent with previous studies showing that imatinib exerts anxiolytic-like effects in different experimental models [48,49]. However, one previous study reported that imatinib may induce depressive-like behavioral changes, which appears inconsistent with our observed anxiolytic-like effect [50]. Such variability may reflect differences in experimental paradigms, dosing regimens, administration routes, or disease models. In addition, different behavioral assays capture distinct dimensions of anxiety. The elevated plus maze (EPM), which relies on approach–avoidance conflict in an elevated environment, may be less sensitive to subtle changes in innate threat processing compared with the light–dark box test [51]. This may explain the lack of significant differences observed in the EPM in the present study. Notably, relatively few studies have directly investigated the behavioral effects of imatinib on anxiety-related paradigms, and the available findings remain limited.

To explore the potential mechanisms underlying these behavioral changes, we performed untargeted metabolomic profiling. Using a relaxed statistical threshold (|log_2_FC| >0.3 with nominal *p* < 0.05), several metabolite features were found to be increased following imatinib treatment, including L-tartaric acid, KAPA, and 8-amino caprylic acid, along with additional features putatively annotated as Alisol B and Nalfurafine. It should be noted that metabolite identification in untargeted metabolomics is primarily based on database matching and therefore remains putative without further structural confirmation, representing an inherent limitation of this approach. In particular, Alisol B is a plant-derived triterpenoid and is unlikely to represent a typical endogenous mammalian metabolite [52]; thus, the detected signal may reflect dietary exposure or a structurally related compound identified through database matching. Despite these limitations, the altered metabolites identified in this study are broadly associated with organic acid, amino acid, and lipid metabolic processes. These metabolic changes may reflect broader metabolic adaptations following imatinib treatment, the functional implications of which are further explored through pathway analysis below.

Imatinib is primarily administered orally and undergoes systemic absorption and hepatic metabolism, with widespread distribution across peripheral tissues [13]. Although its penetration into the central nervous system is relatively limited [53], previous studies have shown that imatinib can indirectly influence brain function through peripheral metabolic and inflammatory pathways [54,55]. In particular, imatinib-mediated modulation of lipid metabolism, cytokine signaling, and gut–brain axis interactions may alter circulating metabolites and signaling molecules, which can subsequently affect central metabolic processes. These pharmacokinetic and systemic effects provide a plausible link between imatinib exposure and the metabolic remodeling observed in the prefrontal cortex.

Metabolomic analysis revealed substantial metabolic differences between the ima and control groups. In particular, multiple sphingolipid-related pathways—including sphingolipid signaling, ceramide synthesis, and sphingolipid metabolism in the endoplasmic reticulum and Golgi apparatus—were significantly enriched. These pathways converge on the ceramide–sphingosine-1-phosphate (S1P) signaling axis, a central regulatory system controlling cellular stress responses, inflammatory signaling, and neuronal communication [56,57,58]. In addition to sphingolipid metabolism, pathway enrichment analysis also revealed alterations in bile acid–sterol metabolic pathways. Bile acids are increasingly recognized not only as regulators of lipid metabolism but also as important signaling molecules linking the gut and the central nervous system [59]. Because bile acid metabolism is strongly influenced by intestinal microbial activity and digestive processes [60], these findings raise the possibility that dietary administration of imatinib may also influence metabolic signaling through the gut–brain axis. Such metabolic changes in the gastrointestinal system could potentially alter circulating metabolites and inflammatory signaling, thereby indirectly affecting neural activity and emotional regulation. Furthermore, enrichment of mitochondrial fatty-acid β-oxidation pathways suggests that imatinib treatment may also influence cellular energy metabolism [61]. Changes in fatty-acid oxidation can affect mitochondrial function, oxidative stress, and lipid-derived signaling molecules [62,63], all of which have been implicated in neuroinflammatory processes and stress-related behaviors.

**Fig. 4.**
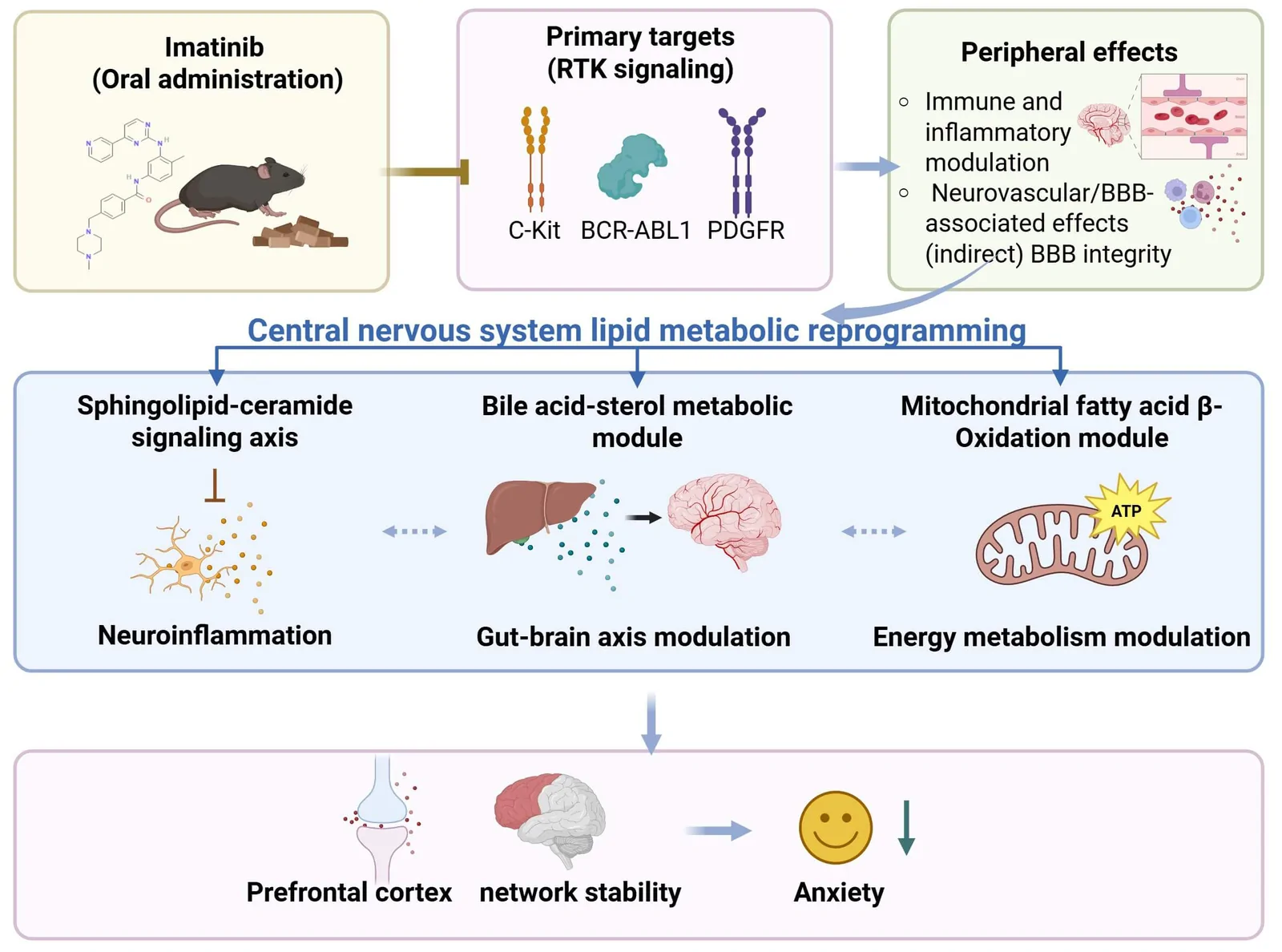
**Proposed mechanisms underlying the anxiolytic-like effects of imatinib**. Imatinib, a receptor tyrosine kinase inhibitor targeting KIT proto-oncogene receptor tyrosine kinase (c-Kit), breakpoint cluster region–Abelson proto-oncogene 1 (BCR-ABL1), and platelet-derived growth factor receptor (PDGFR), may exert peripheral immunomodulatory and neurovascular effects, potentially influencing blood–brain barrier (BBB) function. Metabolomic analysis of the prefrontal cortex (PFC) suggests coordinated alterations in lipid-related pathways, including sphingolipid–ceramide signaling, bile acid–sterol metabolism, and mitochondrial fatty acid β-oxidation. These changes may contribute to the regulation of neuroinflammation and brain energy metabolism, thereby stabilizing PFC network activity and reducing anxiety-like behavior. The mechanisms shown are inferred from metabolomic findings and supported by prior studies, but were not directly tested in this study.

Based on these metabolomic signatures, we propose a model in which inhibition of receptor tyrosine kinase (RTK) signaling by imatinib promotes lipid metabolic reprogramming across multiple lipid-regulatory modules, including the sphingolipid–ceramide signaling axis, bile acid–sterol metabolism, and mitochondrial fatty-acid oxidation. Through these coordinated metabolic adaptations, lipid-mediated inflammatory signaling may be reduced, creating a neurochemical environment that favors stable neuronal activity. Because the prefrontal cortex plays a central role in emotional regulation and stress adaptation, reduced lipid-driven inflammatory signaling within this region may contribute to stabilization of prefrontal cortical networks. Such network stabilization could ultimately underlie the reduced anxiety-like behavior observed in imatinib-treated animals (Fig. 4).

However, several limitations should be acknowledged, including the use of only male mice, the relatively short treatment duration, and the exploratory nature of untargeted metabolomics without targeted validation. Future studies incorporating targeted lipidomics, mechanistic pathway assays, and longitudinal designs will be required to further validate these findings.

Taken together, these findings suggest that imatinib may exert anxiolytic-like effects by modulating RTK-dependent lipid metabolic pathways, thereby reshaping lipid-mediated inflammatory signaling and promoting functional stability of prefrontal cortical circuits.

## 5. Conclusions

In summary, the present study demonstrates that imatinib produces a selective anxiolytic-like effect without altering baseline locomotor activity, as reflected by its behavioral impact in the light–dark box but not in the open field or elevated plus-maze tests. These findings suggest that imatinib may exert context-dependent effects on anxiety-like behavior, particularly in domains related to innate threat processing.

Complementary metabolomic analyses further indicate that imatinib treatment is associated with coordinated alterations in lipid-related pathways, including sphingolipid–ceramide signaling, bile acid–sterol metabolism, and mitochondrial fatty-acid β-oxidation, pointing to a potential role of lipid-mediated inflammatory regulation in linking systemic metabolism to brain function.

Taken together, this study highlights a potential connection between tyrosine kinase inhibitor therapy and brain metabolic regulation, and provides a conceptual framework for understanding how peripheral metabolic adaptations may influence neuropsychiatric outcomes. These findings provide a foundation for future studies exploring the neurobiological and translational significance of targeted cancer therapies.

## Data Availability

The datasets generated during the current study have been deposited in Mendeley Data and are publicly available at: https://data.mendeley.com/datasets/6p9vp3vj3d/1.
