# Supplementary material for: Imatinib-Induced Changes in Anxiety-Like Behaviors are Associated With Prefrontal Cortical Metabolic Remodeling in Mice
Source: Alpha Psychiatry. 2026 Aug 17;27(4):51694. doi: 10.31083/AP51694 (PMC13540018; doi:10.31083/AP51694)
Supplement: Supplementary file 1 [file 2757-8038-27-4-51694-s1.zip › ARRIVE.pdf]

NOTE: Please save this file locally before filling in the table, DO NOT work on the file within your internet browser as changes will not be saved. Adobe Acrobat Reader (available free here) is recommended for completion.

The ARRIVE guidelines 2.0: author checklist

The ARRIVE Essential 10

These items are the basic minimum to include in a manuscript. Without this information, readers and reviewers cannot assess the reliability of the findings.

Item		Recommendation	Section/line number, or reason for not reporting
**Study design**	1	For each experiment, provide brief details of study design including:<br>a. The groups being compared, including control groups. If no control group has been used, the rationale should be stated.<br>b. The experimental unit (e.g. a single animal, litter, or cage of animals).	Secs. 2.2 (p. 3) and 3.1 (p. 5): control chow vs imatinib chow; behavioral tests and PFC metabolomics.<br>Secs. 2.3-2.4 (p. 3): the individual mouse was the behavioral unit; each individual PFC sample was the
**Sample size**	2	a. Specify the exact number of experimental units allocated to each group, and the total number in each experiment. Also indicate the total number of animals used.<br>b. Explain how the sample size was decided. Provide details of any *a priori* sample size calculation, if done.	Fig. 1A legend (p. 4); Tables 1-4 (p. 6): 25 mice initially (ctrl 13, ima 12); one ima mouse excluded; final behavioral n = 13 and 11. Metabolomics n is not explicitly<br>Not reported in the manuscript: sample size was based on previous studies and experimental feasibility; no formal a priori calculation was performed
**Inclusion and exclusion criteria**	3	a. Describe any criteria used for including and excluding animals (or experimental units) during the experiment, and data points during the analysis. Specify if these criteria were established *a priori*. If no criteria were set, state this explicitly.<br>b. For each experimental group, report any animals, experimental units or data points not included in the analysis and explain why. If there were no exclusions, state so.<br>c. For each analysis, report the exact value of *n* in each experimental group.	Sec. 2.1 (pp. 2-3): age-matched male C57BL/6J mice. Behavioral exclusion: values outside the valid range in more than two tests. The manuscript does<br>Not reported in the manuscript:<br>Fig. 1 legend (p. 4) and Tables 1-4 (p. 6): behavioral n = 13 ctrl and n = 11 ima. Metabolomics n is not explicitly [illegible]
**Randomisation**	4	a. State whether randomisation was used to allocate experimental units to control and treatment groups. If done, provide the method used to generate the randomisation sequence.<br>b. Describe the strategy used to minimise potential confounders such as the order of treatments and measurements, or animal/cage location. If confounders were not controlled, state this explicitly.	Sec. 2.2 (p. 3): mice were randomly assigned to groups. The method used to generate the randomisation sequence was not reported.<br>Secs. 2.1-2.3 (pp. 2-3): same-cohort age-matched males and consistent housing/testing conditions. Treatment/test order and cage location were not reported.
**Blinding**	5	Describe who was aware of the group allocation at the different stages of the experiment (during the allocation, the conduct of the experiment, the outcome assessment, and the data analysis).	Not reported in the manuscript: the drug-administration investigator knew allocation; behavioral testing and data analysis were performed by a separate investigator blinded to treatment.
**Outcome measures**	6	a. Clearly define all outcome measures assessed (e.g. cell death, molecular markers, or behavioural changes).<br>b. For hypothesis-testing studies, specify the primary outcome measure, i.e. the outcome measure that was used to determine the sample size.	Secs. 2.3-2.5 (pp. 3-5): OFT distance, center time and entries; LDBT light time and transitions; EPM open-arm entries and time; untargeted PFC<br>No single outcome was used for sample-size calculation.
**Statistical methods**	7	a. Provide details of the statistical methods used for each analysis, including software used.<br>b. Describe any methods used to assess whether the data met the assumptions of the statistical approach, and what was done if the assumptions were not met.	Secs. 2.5.2 and 2.6 (p. 5): Progenesis QI, Majorbio, R/ropls, MetaboAnalyst 5.0 and GraphPad Prism 10; PCA/PLS-DA, t tests, Mann-Whitney U tests<br>Secs. 2.5.2 and 2.6 (p. 5): Shapiro-Wilk test for normality; t test if normal and Mann-Whitney U otherwise; PLS-DA cross-validation/permutation; QC variables
**Experimental animals**	8	a. Provide species-appropriate details of the animals used, including species, strain and substrain, sex, age or developmental stage, and, if relevant, weight.<br>b. Provide further relevant information on the provenance of animals, health/immune status, genetic modification status, genotype, and any previous procedures.	Sec. 2.1 (pp. 2-3): male C57BL/6J mice, 8-10 weeks old. Body weight was not reported.<br>Sec. 2.1 (p. 2): purchased from Speyford (Beijing) Biotechnology and housed under standard conditions. Health/immune status, genotype, genetic
**Experimental procedures**	9	For each experimental group, including controls, describe the procedures in enough detail to allow others to replicate them, including:<br>a. What was done, how it was done and what was used.<br>b. When and how often.<br>c. Where (including detail of any acclimatisation periods).<br>d. Why (provide rationale for procedures).	Secs. 2.2-2.5 (pp. 3-5): imatinib chow (estimated 200 mg/kg/day) vs standard chow, behavioral assays, PFC<br>Secs. 2.2-2.3 (p. 3) and Fig. 1A (p. 4).<br>Secs. 2.1 and 2.3 (pp. 2-3).<br>Introduction and Sec. 2 (p. 2): to determine whether imatinib alters anxiety-like behavior and PFC metabolism
**Results**	10	For each experiment conducted, including independent replications, report:<br>a. Summary/descriptive statistics for each experimental group, with a measure of variability where applicable (e.g. mean and SD, or median and range).<br>b. If applicable, the effect size with a confidence interval.	Sec. 3 (pp. 5-9), Figs. 1-3 and Tables 1-4: behavioral summaries are mean +/- SEM with n and p values; metabolomics findings are reported in the [illegible]<br>Not reported: effect sizes and confidence intervals were not provided. Test statistics and p values are reported in the Fig. 1 legend and Tables 1-4 (pp. 4 and 6).
